# Cosmetic Outcomes and Safety of Hair Transplantation and Topical Minoxidil for Temporal Triangular Alopecia: A Systematic Review

**DOI:** 10.1111/jocd.71140

**Published:** 2026-08-16

**Authors:** Abdulaziz AlRoujayee

**Affiliations:** ^1^ Department of Dermatology, College of Medicine Imam Mohammad Ibn Saud Islamic University (IMSIU) Riyadh Saudi Arabia

**Keywords:** congenital triangular alopecia, cosmetic dermatology, hair transplantation, minoxidil, systematic review, temporal triangular alopecia

## Abstract

**Background:**

Temporal triangular alopecia (TTA) is a non‐scarring alopecia affecting the frontotemporal region, with estimated prevalence of 0.11%. Despite recognized aesthetic and psychological impact, particularly among children and adolescents, no systematic evaluation has synthesized available evidence on its treatment.

**Aims:**

This systematic review synthesizes and evaluates surgical and medical management of TTA, with emphasis on cosmetic outcomes, durability, and safety.

**Methods:**

Following PRISMA 2020, a systematic search was conducted from inception to December 30, 2025 using PubMed, Web of Science, and Cochrane CENTRAL. Eligible studies reported cosmetic outcomes, adverse events, or patient satisfaction with hair transplantation or topical minoxidil for TTA.

**Results:**

Nine studies met inclusion criteria (*n* = 23 patients): three case reports of topical minoxidil and five case reports and one case series of hair transplantation (follicular unit transplantation or excision). Hair transplantation was associated with cosmetic improvement (20 patients, six studies), maintained up to 6 years in one case. Topical minoxidil (three cases) was associated with cosmetic improvement; relapse after discontinuation was reported in one case, with outcomes after discontinuation not assessed in the other two. Both interventions were associated with favorable safety profiles, with no serious adverse events across the included studies.

**Conclusions:**

Topical minoxidil may be a non‐invasive option for pediatric patients or those preferring non‐surgical management, and hair transplantation for adults seeking durable cosmetic correction. These findings reflect low‐level evidence from case reports and a case series (Level IV, Oxford Centre for Evidence‐Based Medicine) and should be considered descriptive observations from a small evidence base. Given the scarce published evidence, higher‐quality prospective studies with longer follow‐up are needed, particularly in pediatric populations.

## 1 Introduction

Temporal triangular alopecia (TTA), also known as congenital triangular alopecia, is a non‐scarring alopecia characterized by a circumscribed triangular or lancet‐shaped patch of fine vellus hairs in the frontotemporal scalp region [1], first described by Sabouraud in 1905 [2]. It is best explained as a focal area of hair miniaturization with vellus hair formation. The prevalence of TTA is approximately 0.11%, although evidence suggests the true prevalence is higher, likely because its clinically benign course leads to underdiagnosis [3]. More than half of cases (55.8%) are diagnosed in childhood, between the ages of two and 9 years, while 36.5% are diagnosed at birth. There is no sex predilection (male 50.9%), with a left‐sided predominance (55.8%) compared with right‐sided (30.8%) or bilateral (13.5%) involvement [1, 4].

Therapeutic approaches for TTA have evolved from conservative reassurance to medical and surgical interventions. Histopathologically, TTA demonstrates preservation of follicular units rather than follicular destruction, a feature that distinguishes it from other types of scarring alopecia. The histological picture is similar to that of androgenetic alopecia, supporting the use of topical minoxidil to stimulate existing hair follicles and also the successful uptake of transplanted follicular units [4].

TTA carries a significant aesthetic and psychological burden, particularly among children and adolescents, in whom the lesion is often visible and may draw unwanted attention in school settings. Additionally, there is growing evidence of hair loss impacting self‐esteem, personality, and social performance [5]. Correspondingly, topical minoxidil and hair transplantation offer both cosmetic restoration and psychological benefits, including improved self‐esteem [5, 6, 7].

Research on TTA is limited, and no prior systematic review has comprehensively evaluated the published evidence on topical minoxidil and hair transplantation for TTA. This gap in the literature leaves clinicians without evidence‐based guidance for treatment selection and aesthetic outcome optimization. The aim of this systematic review is to evaluate the cosmetic efficacy, safety, and durability of topical minoxidil and hair transplantation, including follicular unit transplantation (FUT) and follicular unit excision (FUE), for temporal triangular alopecia.

## 2 Methodology

### 2.1 Study Design and Registration

We conducted this systematic review in accordance with the Preferred Reporting Items for Systematic Reviews and Meta‐Analyses (PRISMA) 2020 guidelines [8]; the completed PRISMA 2020 checklist is provided in Table S1. The protocol was Prospectively Registered with the International Prospective Register of Systematic Reviews (PROSPERO) under registration number CRD420251274131 in December 2025 [9].

### 2.2 Eligibility and Exclusion Criteria

Studies were selected based on the following predetermined criteria, defined according to the PICOS (Population, Interventions, Comparisons, Outcomes, Study Design) framework:

#### 2.2.1 Participants

Adult and pediatric patients (any age) with a clinical diagnosis of TTA.

#### 2.2.2 Interventions

(1) Hair transplantation (FUT or FUE) or (2) topical minoxidil (any concentration, frequency, or duration). Both treatments were evaluated as independent interventions of interest rather than as comparators of one another, owing to the absence of direct comparative studies.

#### 2.2.3 Comparators

Other formal treatments or placebo, if reported. No formal comparator was specified, as direct head‐to‐head studies were absent from the published literature.

#### 2.2.4 Outcomes

##### 2.2.4.1 Primary Efficacy Outcome

Clinically observable improvement in hair coverage of the alopecic patch, as assessed by clinical photography and the investigator's evaluation. No standardized or validated outcome instruments were used across the included studies; outcome assessment was subjective and qualitative.

##### 2.2.4.2 Secondary Outcomes

[1] Durability of cosmetic improvement and relapse following treatment cessation; [2] incidence of treatment‐related adverse events (AEs); [3] serious adverse events; [4] time to first visible response; and [5] patient‐reported satisfaction if reported.

#### 2.2.5 Exclusion Criteria

[1] Review articles, editorial letters without original data; [2] non‐English publications without an available translation; and [3] studies with insufficient outcome data.

### 2.3 Study Design

Randomized controlled trials, observational studies, cohort studies, case series, and case reports.

### 2.4 Information Sources and Search Strategy

A comprehensive, systematic literature search was performed from inception to December 30, 2025 across three major electronic databases: PubMed/MEDLINE, Web of Science Core Collection, and the Cochrane Central Register of Controlled Trials (CENTRAL). The search strategy used combined controlled vocabulary (MeSH in PubMed) and free‐text terms related to three key concepts: [1] temporal triangular alopecia, [2] minoxidil, and [3] hair transplantation. The full search strategy was adapted to the other databases with regard to syntax and subject headings. No language restrictions were applied, and in the interest of identifying additional relevant studies, we manually searched the reference lists of all included studies and relevant systematic reviews. Gray literature sources were not searched. The complete reproducible search strategies for the respective databases, including available MeSH terms, Boolean operators, and free‐text terms, are provided in Table S2.

### 2.5 Study Selection Process

Records obtained from the database searches were imported into Excel (Microsoft Corp, Seattle, WA, USA) and deduplicated using the EndNote reference management software (Clarivate Analytics, Philadelphia, PA, USA). The study selection process was performed independently by two reviewers (A.R. and A.A.) in two stages. First, titles and abstracts were screened against the eligibility criteria. Second, the full texts of potentially relevant studies were retrieved and assessed in detail for final inclusion. Discrepancies between reviewers at either stage were resolved through discussion and consensus; if agreement could not be reached, a third senior reviewer (M.Z.) was consulted. A PRISMA flow diagram was used to document the screening process and reasons for exclusion at the full‐text stage.

### 2.6 Data Extraction and Management

A standardized, pilot‐tested data extraction form was developed in Excel (Microsoft Corp, Seattle, WA, USA) to ensure consistency. Data extraction was performed independently by two reviewers. Extracted data included:

1. Study characteristics: First author, publication year, country, study design, funding source.
2. Participant characteristics: Sample size, age, sex, TTA type, disease duration, lesion location.
3. Intervention details: Drug name, formulation, concentration, application frequency, treatment duration.
4. Outcome data: For each case or patient, the following were extracted: reported treatment response (improved/not improved), method of outcome assessment, follow‐up duration, relapse status, graft survival and subsequent coverage (where applicable), and any adverse events. Given that all the studies included were case reports or case series without comparator arms, no quantitative effect measures (e.g., risk ratios, mean differences) were extracted.
5. Methodological data: Any disagreements in extracted data were resolved by re‐examining the source publication and discussion. Where key data were missing or unclear, we attempted to contact the corresponding authors via email on two separate occasions. If no response was received, the available data were used, and the assumptions were clearly stated in the analysis.

### 2.7 Risk of Bias and Grade Assessment

Methodological quality of case reports and case series was assessed using the Joanna Briggs Institute (JBI) critical appraisal checklists [10]. This assessment is based on eight criteria for case reports and ten criteria for case series. Item responses are yes, no, unclear, or not applicable with an overall appraisal to include or exclude the study. Two reviewers independently graded the studies using the Oxford Centre for Evidence‐Based Medicine (OCEBM) grading system [11]. Discrepancies between the reviewers were resolved by discussion. Formal assessment of reporting bias (e.g., funnel plot analysis) was not feasible given the small number of studies (*n* = 9) and their case‐level design. Therefore, publication bias favoring positive outcomes cannot be excluded, as unsuccessful treatments are less likely to be reported as case reports.

### 2.8 Statistical Analysis

#### 2.8.1 Qualitative Analysis

For qualitative synthesis, study characteristics including design, setting, population demographics, intervention protocols, and outcome measures were extracted into standardized tables using Excel (Microsoft Corp., Seattle, WA, USA). Study‐level summary statistics were extracted as reported by the original authors.

#### 2.8.2 Quantitative Analysis

Treatment outcomes were summarized descriptively using frequency, proportion, and ranges. No quantitative effect measures (e.g., risk ratios, mean differences) were extracted, as the studies included were case reports and case series and did not provide comparator‐arm data suitable for effect size calculation. Given the limited number of observational studies and the substantial methodological and clinical heterogeneity across the included evidence base, a meta‐analysis was not performed.

## 3 Results

**FIGURE 1:**
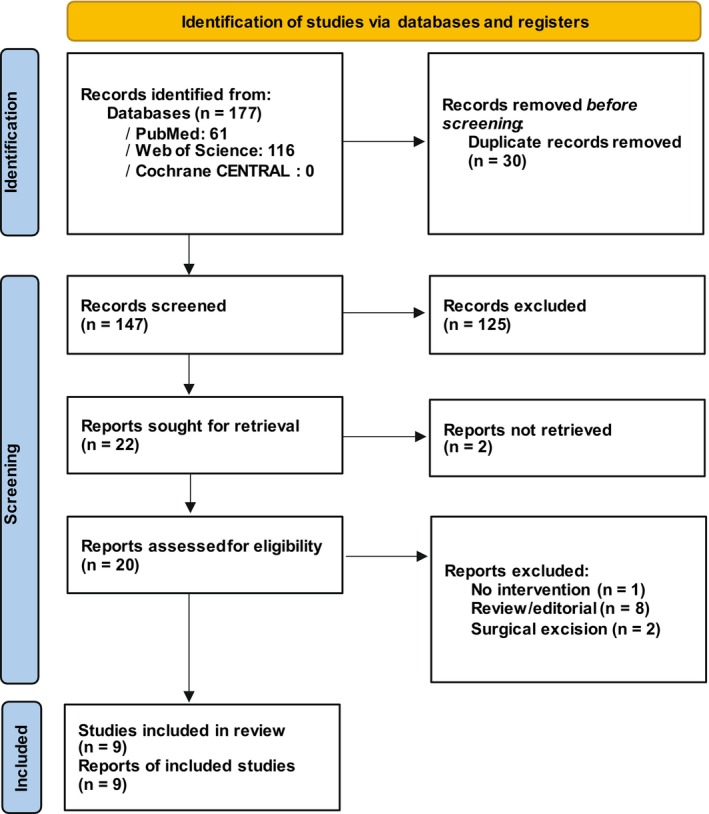
PRISMA flow diagram of study selection for systematic review evaluating topical minoxidil and hair transplantation for temporal triangular alopecia.

Figure 1 presents the PRISMA flow diagram [8] detailing the study selection process for this systematic review evaluating topical minoxidil and hair transplantation for treatment of TTA. A comprehensive literature search across three major databases (PubMed, Web of Science Core Collection, and Cochrane CENTRAL) identified 177 records. After removing 30 duplicates, 147 records were screened, and 22 full text articles were assessed for eligibility (Figure 1). Owing to the rarity of TTA, large‐scale studies are lacking, and most previous research has been case reports or case series. Surgical interventions have been the most frequently used modality in literature. Ultimately, nine studies met the inclusion criteria and were included in the qualitative synthesis. These studies are by Bang et al., Chung et al., Gye et al., Jiménez‐Acosta & Ponce, Pathania, Sutisna et al., Unger & Alsufyani, Vieira & Vieira, and Wu et al. [6, 7, 12, 13, 14, 15, 16, 17, 18].

### 3.1 Study Characteristics

Nine studies were included in this systematic review, comprising eight case reports and one case series, all published between 2009 and 2025. No randomized controlled trials or comparative studies were identified. Three studies investigated topical minoxidil (two using a 5% concentration [7, 12] and one using a 3% concentration [13]), while six evaluated hair transplantation procedures, five using FUT and one case series using FUE [6, 14, 15, 16, 17, 18]. The total sample included 23 patients, three treated with topical minoxidil and 20 with hair transplantation. The characteristics of the included studies are summarized in Table 1. Follow‐up duration ranged from 4 weeks to 6 years, and the included studies were geographically diverse, conducted in South Korea, India, Indonesia, Canada, Spain, the USA, and Brazil.

**TABLE 1:** Characteristics of included studies.

Study ID (Author, Year)	Country	Study design	Total sample size	Intervention	Primary outcome measure	Treatment duration	JBI score	Category
Bang et al. (2013) [13]	South Korea	Case report	1	Minoxidil 3%	Regrowth; relapse on discontinuation	NR	8/8	Medical
Pathania (2020) [12]	India	Case report	1	Minoxidil 5% (twice daily)	Rapid response at 4 weeks; terminal hair growth	4 weeks	7/8	Medical
Sutisna et al. (2024) [7]	Indonesia	Case report	1	Minoxidil 5% (twice daily)	Terminal hair at 2 months; dense regrowth at 8 months	8 months	8/8	Medical
Wu et al. (2009) [16]	Canada	Case report	1	FUT (325 FU)	Cosmetically satisfying results	16 months	8/8	Surgical
Jiménez‐Acosta & Ponce (2009) [15]	Spain	Case report	1	FUT (250 FU)	Excellent result	2 years	6/8	Surgical
Unger & Alsufyani (2011) [14]	USA	Case report	1	FUT (1449 FU)	Stable at 6 years (longest follow‐up)	6 years	8/8	Surgical
Chung et al. (2012) [6]	South Korea	Case report	1	FUT (987 FU)	Excellent outcome	18 months	8/8	Surgical
Gye et al. (2013) [17]	South Korea	Case report	1	FUT (270 FU)	75% coverage	15 months	8/8	Surgical
Vieira & Vieira (2025) [18]*	Brazil	Case series	15	FUE (30–55 FU/cm^2^)	Satisfactory outcomes	12 months	7/10	Surgical

Abbreviations: FUE, Follicular unit excision; FUT, Follicular unit transplantation; JBI, Joanna Briggs Institute; NR, Not reported.

* Vieira & Vieira [18] reported total data for the case series (*n* = 15); individual‐level demographic data were not separately reported and are therefore excluded from per‐patient demographic analyses.

### 3.2 Participant and Disease Characteristics

A total of 23 patients were represented across the nine included studies. Sample size ranged from one patient in eight case reports [6, 7, 12, 13, 14, 15, 16, 17] to 15 patients in one case series [18]. All studies enrolled patients with TTA. Individual‐level demographic data were available for eight patients across eight studies; the case series by Vieira & Vieira [18] reported only total data and was therefore not included in patient‐level demographic summaries. The mean age of participants, was 10.3 years (range 1–16 years) in the topical minoxidil group, with all being pediatric (under 18 years) [7, 12, 13], versus 21.8 years (range 17–27 years) in the hair transplantation group [6, 14, 15, 17, 18]. Gender distribution showed a male predominance in both groups (67% in the topical minoxidil group and 60% in the hair transplantation group). Lesion location was primarily the left frontotemporal region (62.5% of cases) and the right frontotemporal region (25%), with one bilateral case (12.5%). Associated comorbidities were rare, with one case of phakomatosis pigmentovascularis. Baseline patient characteristics are presented in Table 2 and summarized by treatment group in Table 3.

**TABLE 2:** Participant and disease baseline characteristics.

Study ID (Author, Year)	Age (years)	Gender distribution	Lesion location	Lesion size	Treatment	Adverse events
Bang et al. (2013) [13]	1	Female	Right frontotemporal	2.5 × 1 cm	Minoxidil 3%	Skin irritation (temporary stop)
Pathania (2020) [12]	16	Male	Left frontotemporal	4.5 × 2.5 cm	Minoxidil 5% (twice daily)	None reported
Sutisna et al. (2024) [7]	14	Male	Right frontotemporal	9 × 6 cm	Minoxidil 5% (twice daily)	None reported
Wu et al. (2009) [16]	17	Male	Left frontotemporal	3.5 × 2 cm	FUT (325 FU)	None reported
Jiménez‐Acosta & Ponce (2009) [15]	18	Female	Left frontotemporal	8 cm^2^	FUT (250 FU)	None reported
Unger & Alsufyani (2011) [14]	25	Male	Bilateral on both temples	Left 8 × 5 cm and Right 8 × 4 cm	FUT (1449 FU)	None reported
Chung et al. (2012) [6]	22	Female	Left frontotemporal	None reported	FUT (987 FU)	Mild scalp erythema
Gye et al. (2013) [17]	27	Male	Left frontotemporal	4.5 × 3.2 cm	FUT (270 FU)	Temporary itching and erythema resolved without treatment
Vieira & Vieira (2025) [18]	NR	NR	NR	NR	FUE (30–55 FU/cm^2^)	None reported

Abbreviations: FUE, Follicular unit excision; FUT, Follicular unit transplantation; NR, Not reported.

**TABLE 3:** Baseline patient characteristics by treatment group.

Characteristic	Topical minoxidil (*n* = 3)^a^	Hair transplantation (*n* = 5)^a^	Total (*n* = 8)^a^
Age, years			
Mean (range)	10.3 (1–16)	21.8 (17–27)	17.5 (1–27)
Pediatric (≤ 18 years)	3 (100%)	1 (20%)	4 (50%)
Sex			
Male	2 (67%)	3 (60%)	5 (62.5%)
Female	1 (33%)	2 (40%)	3 (37.5%)
Lesion location			
Left frontotemporal	1 (33%)	4 (80%)	5 (62.5%)
Right frontotemporal	2 (67%)	0 (0%)	2 (25%)
Bilateral	0 (0%)	1 (20%)	1 (12.5%)

^a^ Demographic data exclude the 15 patients in the Vieira & Vieira [18] case series, wherein individual characteristics are not reported. Outcome data elsewhere in the manuscript include all 23 patients across the nine included studies.

### 3.3 Intervention Details

The interventions evaluated were topical minoxidil and hair transplantation including FUT and FUE. Among the minoxidil studies, formulations included 3% and 5% solutions applied twice daily. All minoxidil regimens required continuous application, with treatment durations ranging from 4 weeks to 8 months [12, 13]. For hair transplantation, FUT was performed in five studies using a variety graft quantities [6, 14, 15, 16, 17]. Gye et al. [17] reported one patient with prior surgical excision, which was associated with lower graft uptake owing to previous scarring. Vieira & Vieira [18] evaluated FUE in a case series using the Implantation Coverage Value (ICV) model (30–55 FU/cm^2^). The characteristics of the studies are summarized in Table 1.

### 3.4 Efficacy Outcomes

Outcome assessment across all included studies was based on subjective clinical evaluation and photographic documentation; no validated instruments for hair coverage measurement were reported, which limits objectivity and comparability.

#### 3.4.1 Primary Treatment Endpoints

Across all included studies, the primary efficacy measure was cosmetic improvement, assessed by clinical photography and investigator evaluation (Tables 4 and 5). Of patients treated with topical minoxidil, all three cases demonstrated visible hair regrowth. Pathania [12] reported transition from vellus to terminal hairs within 4 weeks of initiating 5% minoxidil twice daily. Sutisna et al. [7] documented dense hair regrowth for 8 months in a pediatric patient with a large (9 × 6 cm) lesion. Bang et al. [13] similarly reported hair regrowth with 3% minoxidil; treatment discontinuation in this single case was reported to be followed by relapse; the remaining two case reports (Pathania [12]; Sutisna et al. [7]) did not evaluate or report outcomes after discontinuation. Given the very limited sample size (*n* = 3) and heterogeneous follow‐up across the included minoxidil studies, these findings should be regarded as preliminary and hypothesis‐generating rather than as evidence of a generalizable need for continuous therapy.

**TABLE 4:** Descriptive summary of reported outcomes for hair transplantation in Temporal Triangular Alopecia (*n* = 20 patients across 6 studies) [6, 14, 15, 16, 17, 18].

Outcome	Hair transplantation (FUT/FUE)
Number of patients	20 patients across 6 studies (case reports/series)
Reported outcomes	Qualitative cosmetic improvement in all reported cases (20/20)
Durability	Yes; no relapse documented within available follow‐up
Time to visible result	Approximately 12–18 months for graft growth
Reported adverse events	Mild erythema (10%); no serious adverse events reported
Invasiveness	Yes; surgical procedure (FUT or FUE)
Pediatric suitability	Generally postponed until adolescence
Limitations of the evidence	Mainly case reports and case series; no controls, subjective outcomes, variable follow‐up

**TABLE 5:** Descriptive summary of reported outcomes for topical Minoxidil in temporal triangular alopecia (*n* = 3 patients across 3 case reports) [7, 12, 13].

Outcome	Topical minoxidil
Number of patients	3 patients across 3 studies (case reports)
Reported outcomes	Initial cosmetic improvement in all reported cases (3/3). Relapse after discontinuation: reported in one case; not evaluated or not reported in the remaining cases
Durability	Durability after discontinuation was assessed in only one of three cases, in which relapse was reported. The remaining two cases did not evaluate post discontinuation outcomes. The need for continuous application remains a clinical inference rather than an established finding
Time to visible result	Approximately 4 weeks to 8 months
Reported adverse events	Skin irritation reported in approximately one‐third of cases (1/3); no systemic adverse events reported
Invasiveness	Non‐invasive topical therapy
Pediatric suitability	Suitable in pediatric patients in available cases
Limitations of the evidence	Small sample, no standardized outcomes, heterogeneous concentrations

Among hair transplantation recipients (FUT and FUE), all 20 patients across the six included studies reported favorable cosmetic correction, with natural‐appearance hair growth consistently documented; coverage ranged from 75% to 100%. The single suboptimal outcome was reported by Gye et al. [17] wherein a patient with prior surgical excision achieved only 75% improvement; this was attributed to recipient site scarring that compromised graft uptake. The largest study, by Vieira & Vieira [18], reported favorable cosmetic outcomes across 15 patients treated with FUE using the ICV model.

#### 3.4.2 Secondary and Exploratory Efficacy Measures

##### 3.4.2.1 Time to Response

Topical minoxidil demonstrated an earlier onset of visible improvement, with responses observed as early as 4 weeks [12] to 2 months [7]. In contrast, hair transplantation required a longer duration, with final cosmetic results typically achieved at 12 [6] to 18 months [18]; additional time post‐procedure is inherently required for the graft cycle to complete and terminal hair to grow.

##### 3.4.2.2 Durability and Maintenance

Hair transplantation was associated with durable results across the included studies, with follow‐up extending to 6 years [14]. Relapse after discontinuation was documented in one of the three minoxidil case reports (Bang et al. [13]); the remaining two case reports did not evaluate treatment durability after discontinuation. The available evidence is therefore insufficient to estimate the frequency of relapse after discontinuation of topical minoxidil for TTA.

##### 3.4.2.3 Graft Survival and Coverage

Across the included hair transplantation studies, reported graft survival and resulting coverage were high, with coverage ranging from 75% to 100%. Using the ICV model (30‐55 FU/cm^2^), Vieira & Vieira [18] achieved uniform coverage across varying lesion sizes, suggesting that standardized coverage targets may optimize surgical planning and outcomes.

##### 3.4.2.4 Patient‐Reported Outcomes

Qualitative narrative reports in the included studies described high patient satisfaction following both interventions, particularly after hair transplantation. No validated patient‐reported outcome measure was used in any study; satisfaction data should therefore be interpreted as investigator‐summarized rather than formally measured.

### 3.5 Safety and Tolerability

#### 3.5.1 Adverse Event Profiles (AEs)

With topical minoxidil, local application site reactions were the most common adverse events (AEs). Specifically Bang et al. [13] reported skin irritation leading to temporary treatment discontinuation; the patient tolerated the medication well upon restarting treatment. The other minoxidil studies did not report any local AEs [7, 12]. For hair transplantation, transient scalp erythema at the recipient site occurred in 10% of patients (2/20) [6, 17], resolving spontaneously within days post‐procedure. No donor site complications, infections, or graft‐related AEs were documented. Serious AEs were absent across all studies, and no treatment‐related systemic effects were reported in either group (Table 2).

#### 3.5.2 Discontinuations

Treatment discontinuation due to AEs was rare among patients treated with minoxidil. The only documented discontinuation was reported by Bang et al. [13], in which a patient temporarily discontinued therapy due to skin irritation; the patient subsequently tolerated restarting treatment. For hair transplantation, no patients discontinued follow‐up due to procedure‐related complications.

### 3.6 Risk of Bias and Certainty of Evidence Assessment

Risk of bias assessment using the JBI critical appraisal checklists for case reports and case series yielded favorable results. However, methodological limitations of case‐level evidence were present across all studies, including the absence of control groups and variable follow‐up durations. The case series by Vieira & Vieira [18] had a standardized outcome assessment. All studies were included based on critical appraisal findings (Table 1). Grading with the OCEBM system, the certainty of evidence for TTA treatment outcomes was Level IV, as all included studies were case reports and non‐comparative case series with small sample sizes.

### 3.7 Summary of Findings

The synthesized evidence from the nine observational studies indicates that both topical minoxidil and hair transplantation were associated with reported cosmetic improvement in the included cases. Hair transplantation was associated with durable results in the available case‐level data, with outcomes maintained for up to 6 years of follow‐up. In the very limited topical minoxidil evidence base (*n* = 3 patients across three case reports, with non‐standardized outcome assessment and heterogeneous follow‐up), relapse after discontinuation was reported in one case; the remaining two cases did not evaluate outcomes after treatment cessation; these findings should be regarded as preliminary and hypothesis‐generating rather than as definitive evidence regarding the durability of topical minoxidil for TTA. Topical minoxidil notably offered a faster onset of visible hair improvement, reported as early as 4 weeks [12], whereas hair transplantation required a longer duration to mature, with final cosmetic results typically at 12–18 months [6, 18]. The safety profiles of both interventions appeared favorable, with only minor local adverse events reported in 33% of topical minoxidil cases and 10% of hair transplantation cases; no serious adverse events were documented in either group. Discontinuation due to AEs was rare; one case of temporary minoxidil discontinuation due to local irritation was reported. The evidence reports cosmetic improvement following both interventions; however, this evidence is limited by the absence of randomized controlled trials, small sample sizes, and the predominance of case‐level evidence. The reported outcomes for hair transplantation and topical minoxidil are summarized descriptively in Tables 4 and 5, respectively.

## 4 Discussion

This systematic review synthesized the available evidence evaluating topical minoxidil and hair transplantation (FUT/FUE) for temporal triangular alopecia, encompassing nine included studies (23 patients). Both interventions were associated with cosmetic improvement in the reported cases. Hair transplantation was associated with sustained long‐term correction, with follow‐up extending up to 6 years [14]. However, within the limited topical minoxidil evidence (*n* = 3), relapse after discontinuation was documented in only one case [13], while the other two did not evaluate outcomes after treatment cessation [7, 12]. These case‐level observations should be regarded as preliminary.

TTA lesions contain miniaturized vellus hair follicles rather than destroyed follicles, a histologic pattern similar to that of androgenetic alopecia; this may explain both the success of pharmacologic stimulation and the stable long‐term outcomes after hair transplantation [19].

The Implantation Coverage Value (ICV) model proposed by Vieira & Vieira [18], which suggests standardized coverage targets of 30–55 FU/cm^2^, offers an exploratory framework for graft coverage planning; however, it was derived from a single case series and requires validation in larger, independent studies. Notably, congenital TTA is reported to require a higher ICV than acquired TTA, which may reflect an early predominance of miniaturized vellus follicles in congenital lesions that results in reduced effective follicular coverage.

The clinical implication of this systematic review on TTA is in the selection decision between durable correction and avoiding invasive surgery. Topical minoxidil provides rapid visible improvement and avoids procedural risks but requires maintenance therapy with prolonged application. Hair transplantation provides a one‐time structural correction with durable results, but also features costs associated with the surgery, donor site considerations, and a delay to maximal cosmetic outcome. None of the included studies reported the use of topical minoxidil as maintenance therapy following hair transplantation. In routine clinical practice for androgenetic alopecia, topical minoxidil is prescribed post‐transplantation to support graft survival and prevent further follicular miniaturization. The absence of comparable data in the TTA literature represents a notable gap, and future studies evaluating combined protocols are warranted.

The safety profiles of both topical minoxidil and hair transplantation are encouraging, with no serious adverse events reported. Among the three patients treated with topical minoxidil, mild scalp irritation occurred in one (33%) and resolved without treatment modification or discontinuation. Notably, no systemic effects such as cardiovascular symptoms, hypertrichosis, or contact dermatitis were reported; however, the limited sample size and short follow‐up period prevent firm conclusions about the long‐term safety of topical minoxidil in pediatric populations.

For hair transplantation, transient scalp erythema at the recipient site occurred in 10% of patients (2/20) and resolved spontaneously within days. No donor‐site complications, wound infections, folliculitis, or graft‐related adverse events were documented. Overall, the current evidence suggests that both interventions are well tolerated when properly selected according to patient age and clinical context; however, prospective studies with systematic adverse‐event monitoring are needed to fully characterize the safety profiles of these treatments.

**FIGURE 2:**
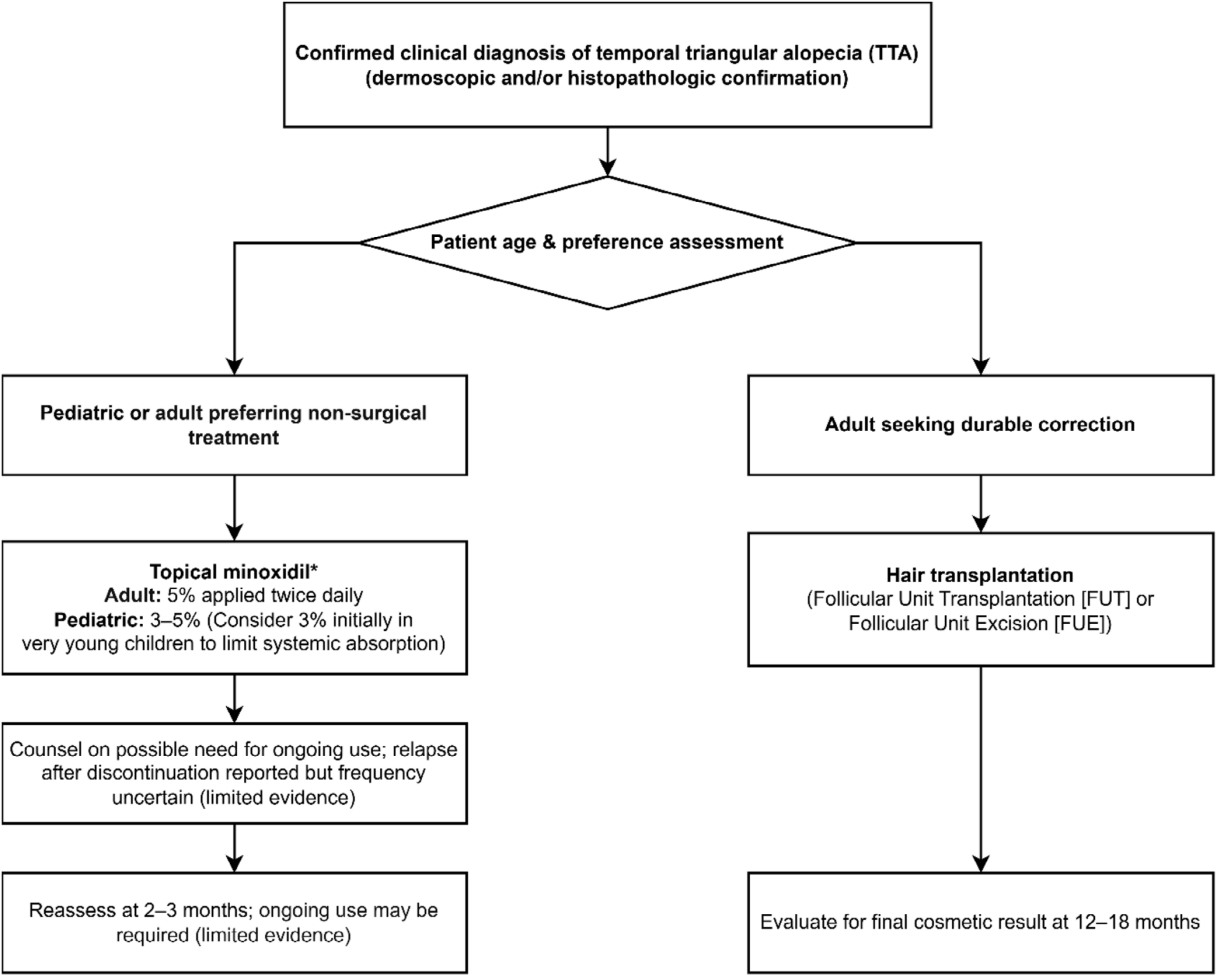
Proposed clinical management algorithm for temporal triangular alopecia (TTA). *Topical minoxidil (adult 5%; pediatric 3%–5%); see the Discussion section for application details and supporting evidence. The level of evidence is Level IV according to the Oxford Centre for Evidence‐Based Medicine (OCEBM), as these recommendations are derived from case reports and non‐comparative case series. Recommendations are intended as supportive clinical aid rather than formal guidelines. Treatment decisions should always be guided by individualized clinical judgment.

To synthesize our findings and provide a practical tool for clinicians, we propose a clinical decision‐making algorithm for the management of TTA (Figure 2). These recommendations are based on age and patient preference. For adults seeking durable cosmetic correction, hair transplantation (FUT or FUE) may be considered, with evaluation of the final cosmetic result at approximately 12–18 months. For adults preferring non‐surgical management, topical minoxidil 5% twice daily is reasonable, with counseling regarding the need for continued application. For pediatric patients, topical minoxidil was applied at concentrations of 3%–5% across included cases; the lower concentration (3%) may be considered initially in very young children as a precautionary measure, given potential for greater systemic absorption relative to body size [20]. Treatment response should be reassessed at approximately 2–3 months, and patients counseled that relapse after discontinuation has been reported and may occur; the available evidence is insufficient to estimate its frequency, and the need for long‐term maintenance therefore remains a clinical inference. In pediatric patients, surgical intervention is generally postponed until late adolescence, when lesion stability and patient cooperation permit, at which point hair transplantation may be considered if durable correction is desired. These recommendations correspond to OCEBM Level IV evidence, as they are derived from case reports and non‐comparative case series, and should be guided by individualized clinical judgment.

This systematic review represents the first comprehensive evaluation of TTA treatments, applying the PICOS framework and following the PRISMA 2020 checklist to assess topical minoxidil and hair transplantation for TTA. Nevertheless, several limitations are evident and largely reflect the rarity of TTA, such as the lack of randomized controlled trials; small sample size, which can limit generalizability; the absence of direct comparative studies; and potential publication bias favoring positive outcomes. These constitute potential research gaps for further studies to strengthen these recommendations.

## 5 Conclusions

This systematic review evaluated topical minoxidil and hair transplantation for temporal triangular alopecia. The choice between these treatments is based on the patient's age and clinical evaluation. For clinicians, the available evidence suggests that topical minoxidil may be an appropriate option for pediatric populations or for those preferring non‐invasive management; however, relapse after discontinuation has been reported in one case, and the available evidence is insufficient to estimate its frequency; ongoing application may be required, but this remains a clinical inference rather than an established finding. Hair transplantation may be a practical option for adult patients seeking durable cosmetic correction. Both interventions showed favorable safety profiles, with only minor local adverse events and no serious adverse events reported. Given the rarity of TTA and the predominance of case reports and case series in the current literature, the conclusions of this review have a low level of evidence and require validation. High‐quality prospective studies with longer follow‐up are needed, particularly in pediatric populations, to strengthen this evidence base.

## Funding

This work was supported and funded by the Deanship of Scientific Research at Imam Mohammad Ibn Saud Islamic University (IMSIU) (grant number IMSIU‐DDRSP2601).

## Ethics Statement

The author confirms adherence to the journal's ethical policies. No ethical approval was required for this systematic review article as data was synthesized from previously published studies.

## Consent

The author has nothing to report.

## Conflicts of Interest

The author declares no conflicts of interest.

## Supporting information


**Table S1:** PRISMA 2020 Checklist (submitted as a separate supplementary file).

**Table S2:** Search strategies used in each database (submitted as a separate supplementary file).

## Data Availability

The data that support the findings of this study are available in the Supporting Information of this article.
