# Supplementary material for: Cosmetic Outcomes and Safety of Hair Transplantation and Topical Minoxidil for Temporal Triangular Alopecia: A Systematic Review
Source: J Cosmet Dermatol. 2026 Aug 16;25(8):e71140. doi: 10.1111/jocd.71140 (PMC13478358; doi:10.1111/jocd.71140)
Supplement: Supplementary file 1 [file JOCD-25-e71140-s001.docx]

**Supplementary Table S2. Search strategies used in each database**

All searches were run from database inception through 30 December 2025. No language restrictions were applied. Reference lists of all included studies and relevant reviews were additionally screened by hand to identify any further eligible records.

**Records identified by database**

| **Database** | **Records identified** |
| --- | --- |
| PubMed/MEDLINE | 61 |
| Web of Science Core Collection | 116 |
| Cochrane CENTRAL | 0 |
| **Total records identified** | **177** |

**1. PubMed / MEDLINE**

**Search date:** December 2025 | Records identified**:** 61

Concept 1 — Condition (TTA terminology)

Concept 2 — Minoxidil

Concept 3 — Hair transplantation

Final is combination

| **Line** | **Search query** |
| --- | --- |
| #1 | "temporal triangular alopecia"[tiab] |
| #2 | "congenital triangular alopecia"[tiab] |
| #3 | "triangular alopecia"[tiab] |
| #4 | (triangular[tiab] AND alopecia[tiab] AND (congenital[tiab] OR temporal[tiab])) |
| #5 | #1 OR #2 OR #3 OR #4 |
| #6 | "Minoxidil"[MeSH] |
| #7 | minoxidil[tiab] |
| #8 | #6 OR #7 |
| #9 | "Hair Transplantation" |
| #10 | "hair transplant*"[tiab] |
| #11 | "follicular unit transplant*"[tiab] OR "FUT"[tiab] |
| #12 | "follicular unit excision"[tiab] OR "follicular unit extraction"[tiab] OR "FUE"[tiab] |
| #13 | "hair restoration"[tiab] |
| #14 | "hair graft*"[tiab] |
| #15 | #9 OR #10 OR #11 OR #12 OR #13 OR #14 |
| #16 | #8 OR #15 |
| #17 | #5 AND #16 |

**2. Web of Science Core Collection**

**Search date:** December 2025 (Records identified**:** 116)

**Search query**

("congenital triangular alopecia" OR "temporal triangular alopecia" OR "Brauer nevus" OR "Brauer's nevus") AND ("treatment" OR "therapy" OR "management" OR "hair transplant*" OR "minoxidil" OR "surgical" OR "FUE" OR "FUT")

**3. Cochrane CENTRAL**

**Search date:** December 2025 (Records identified: 0)

**Search query**

(temporal triangular alopecia OR congenital triangular alopecia) AND (hair transplant* OR follicular unit transplant* OR FUT" OR follicular unit excision" OR follicular unit extraction OR FUE OR hair restoration OR hair graft* OR minoxidil)
